# Characterization of a Mouse Model of Alzheimer’s Disease Expressing Aβ4-42 and Human Mutant Tau

**DOI:** 10.3390/ijms22105191

**Published:** 2021-05-14

**Authors:** Silvia Zampar, Oliver Wirths

**Affiliations:** Department of Psychiatry and Psychotherapy, University Medical Center (UMG), Georg-August-University, D-37075 Göttingen, Germany; silvia.zampar@med.uni-goettingen.de

**Keywords:** Alzheimer’ disease, amyloid β, tau, behavior, neuron loss, transgenic mice, Aβ4-42

## Abstract

The relationship between the two most prominent neuropathological hallmarks of Alzheimer’s Disease (AD), extracellular amyloid-β (Aβ) deposits and intracellular accumulation of hyperphosphorylated tau in neurofibrillary tangles (NFT), remains at present not fully understood. A large body of evidence places Aβ upstream in the cascade of pathological events, triggering NFTs formation and the subsequent neuron loss. Extracellular Aβ deposits were indeed causative of an increased tau phosphorylation and accumulation in several transgenic models but the contribution of soluble Aβ peptides is still controversial. Among the different Aβ variants, the N-terminally truncated peptide Aβ_4–42_ is among the most abundant. To understand whether soluble Aβ_4–42_ peptides impact the onset or extent of tau pathology, we have crossed the homozygous Tg4–42 mouse model of AD, exclusively expressing Aβ_4–42_ peptides, with the PS19 (P301S) tau transgenic model. Behavioral assessment showed that the resulting double-transgenic line presented a partial worsening of motor performance and spatial memory deficits in the aged group. While an increased loss of distal CA1 pyramidal neurons was detected in young mice, no significant alterations in hippocampal tau phosphorylation were observed in immunohistochemical analyses.

## 1. Introduction

Alzheimer’s disease (AD) is a progressive neurodegenerative disorder that is histopathologically characterized by the deposition of extracellular senile plaques containing amyloid-β (Aβ) protein [1], as well as the intracellular accumulation of so-called neurofibrillary tangles (NFTs) consisting of hyperphosphorylated protein tau [2]. While Aβ peptides are derived from proteolytical cleavage events of the transmembrane amyloid precursor protein (APP), tau proteins are brain-specific microtubule-associated molecules enriched in axons. In recent years, a variety of studies employing mainly transgenic AD mouse models investigated the relationship between both pathological hallmarks. According to the amyloid cascade hypothesis, a disequilibrium of Aβ production and clearance is regarded as an upstream event entailing the formation of NFTs and the subsequent loss of synapses and neurons [3]. The common belief is that Aβ acts as a pathological trigger, with tau being the required executor [4,5]. APP transgenic mice expressing the E693Δ mutation display age-dependent accumulation of intraneuronal Aβ oligomers in the absence of amyloid deposits but show abnormal tau phosphorylation from 8 months [6]. Such data is corroborated by studies from double-transgenic mouse models expressing both mutant APP and tau transgenes, showing in general increased levels of phosphorylated tau as well as accelerated tau pathology compared to their single tau transgenic parental strains [7,8,9,10,11,12,13]. However, the importance of tau on the detrimental effect of Aβ is a matter of debate. In many experimental models, tau appears to be essential for the execution of Aβ toxicity as the absence of the endogenous protein seems to confer protection from Aβ-induced neurodegeneration. Indeed, primary neurons isolated from tau knock-out mice resist exposure to an excess of fibrillary Aβ peptides [14]. Hippocampal neuron loss observed in 5XFAD transgenic mice could be rescued 5XFAD on a tau knock-out background, together with an ~50% reduction in amyloid plaque burden [15]. In good agreement, the absence of tau prevented Aβ-induced impairment of long-term potentiation (LTP) in acute slice preparations, suggesting that tau is required for Aβ to impair hippocampal synaptic plasticity [16]. On the other hand, a more recent finding points to the opposite direction. In TgAPP mice, the lack of endogenous tau indicated that synaptic and memory impairments induced by Aβ were tau-independent, as tau suppression did not protect against Aβ-induced deficits in long-term synaptic plasticity and memory or amyloid deposition [17].

While there is convincing evidence that the presence of extracellular Aβ deposits contributes to an increased tau phosphorylation and accumulation, it is less clear if soluble Aβ peptides influence tau pathology in a comparable manner. It has been demonstrated that soluble Aβ dimers isolated from human AD brain induce tau hyperphosphorylation at AD-relevant epitopes when they were applied to hippocampal neurons in sub-nanomolar concentrations [18]. Intracerebral injection of either Aβ_1–40_ or Aβ_1–42_ into P301S tau transgenic mice induced tau phosphorylation and aggregation only in the latter case, while injections of Aβ_1–40_ even decreased tau phosphorylation at certain epitopes [19]. On the other hand, a mouse model expressing human 4-repeat tau protein did not show significant amounts of tau pathology at 6 months of age and co-expression of Aβ_1–42_ in the absence of APP overexpression did not lead to an aggravated phenotype [20]. We have recently generated a transgenic mouse model (Tg4–42) that overexpresses Aβ_4–42_ peptides under the control of the murine neuron-specific Thy1-promoter [21]. Aβ_4–42_ peptides are among the first Aβ peptides that have been identified in human AD brain and represent a highly abundant peptide species [1,22,23]. Tg4–42 mice do not form overt extracellular Aβ deposits but present robust behavioral deficits together with abundant CA1 neuron loss at an age of 6 months [24,25].

Here, we determined whether soluble N-terminally truncated Aβ_4–42_ peptides have an impact on the onset or extent of tau pathology in the absence of confounding human APP overexpression. We generated double-transgenic mice by crossbreeding homozygous Tg4–42 mice with the widely used PS19 tau transgenic model overexpressing tau with the P301S mutation [26] and performed behavioral and neuropathological analysis at different time points.

## 2. Results

### 2.1. Transgene Expression and Weight Assessment in PS19/Tg4–42^hom^

**Figure 1:**
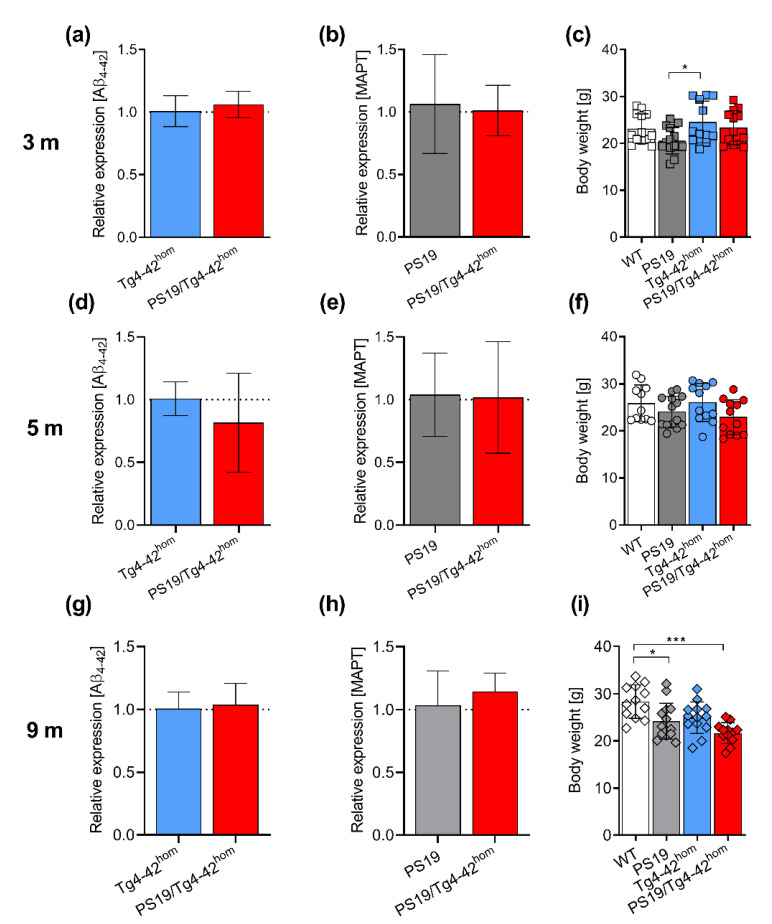
Transgene expression and body weight in PS19/Tg4–42^hom^ mice. Hippocampal mRNA expression of the Aβ_4–42_ transgene (**a**,**d**,**g**) and human PS19 (MAPT) gene (**b**,**e**,**h**) were measured in PS19/Tg4–42^hom^ mice and compared to the ones of the parental lines (*n* = 5–6). At the considered ages of 3 (**c**) or at 5 months (**f**), no changes in body weight were detected in the transgenic lines compared to WT controls, nor in PS19/Tg4–42^hom^ compared to PS19 or Tg4–42^hom^ mice (*n* = 12–14). At 9 months, the body weight of PS19 and PS19/Tg4–42^hom^ mice was significantly reduced compared to WT controls (**i**). All data are given as mean ± SD. (**a**,**b**,**d**,**e**,**g**,**h**) Mann–Whitney test, (**c**,**f**) One-way ANOVA followed by Bonferroni’s multiple comparison: * *p* < 0.05, *** *p* < 0.001.

Transgene mRNA expression levels in the bigenic PS19/Tg4–42^hom^ line were measured and compared to the parental lines at 3, 5 and 9 months of age to exclude an impact of altered transgene expression as a consequence of the crossing (primers listed in Table 1). As Aβ_4–42_ is predominantly expressed in the CA1 region in Tg4–42^hom^ mice [21], hippocampal mRNA extracts were used for the analysis of both transgenes. No significant differences were found in either Aβ_4–42_ transgene expression between Tg4–42^hom^ and PS19/Tg4–42^hom^ (Figure 1a,d,g), or in human MAPT mRNA levels between PS19 and PS19/Tg4–42^hom^ (Figure 1b,e,h) at any time point. Body weight of the transgenic lines did not differ compared to WT littermates, and no significant changes were observed between PS19/Tg4–42^hom^ and the two parental lines at 3 and 5 months (Figure 1c,f). In the aged group, both PS19 and PS19/Tg4–42^hom^ mice revealed a significantly reduced body weight compared to WT mice (*p* < 0.05 for PS19, *p* < 0.001 for PS19/Tg4–42^hom^) (Figure 1i).

### 2.2. PS19/Tg4–42^hom^ Mice Display a Partial Worsening of Motor Performance at 5 Months of Age

**Figure 2:**
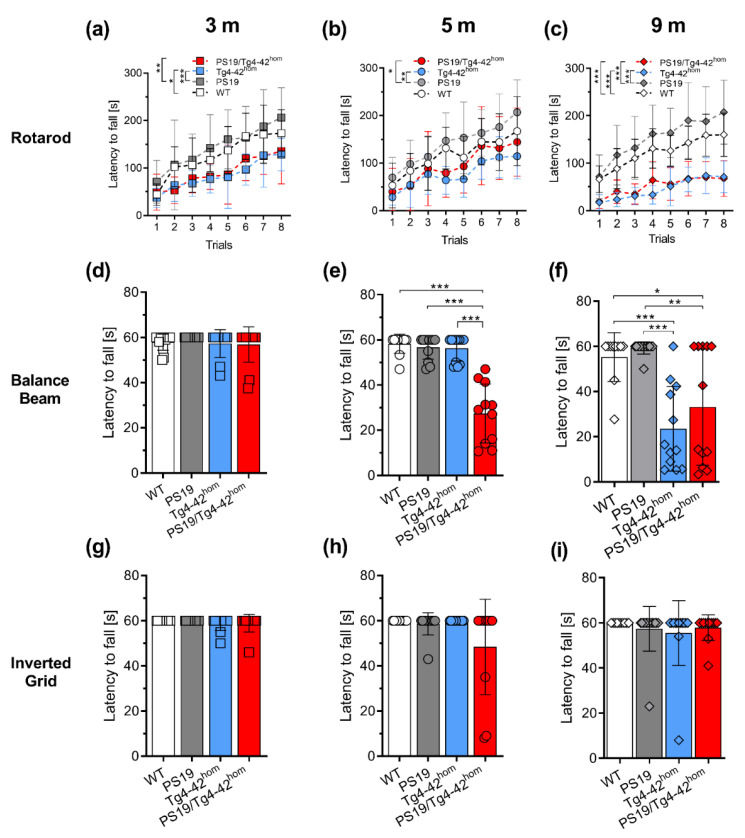
Partial worsening of motor performances in 5-month-old PS19/Tg4–42^hom^ mice. Female and male WT, PS19, Tg4–42^hom^ and PS19/Tg4–42^hom^ mice were tested in the accelerating rotarod (**a**–**c**), balance beam (**d–f**) and inverted grid (**g–i**) tasks at 3, 5 and 9 months of age (n = 12–14). Motor deficits in the accelerating rotarod task were observed in both Tg4–42^hom^ and PS19/Tg4–42^hom^ mice (**a–c**), while only the bigenic line showed a significant reduced latency to fall from the balance beam at 5 months (**e**). At 9 months, both Tg4–42^hom^ and PS19/Tg4–42^hom^ mice revealed a deficit (**f**). No significant differences were observed in the inverted grid task at any age (**g**–**i**). All data are given as mean ± SD. (**a**–**c**) Two-way ANOVA RM, followed by Bonferroni’s multiple comparison, (**d**–**i**) One-way ANOVA, followed by Bonferroni’s multiple comparison: * *p* < 0.05, ** *p* < 0.01, *** *p* < 0.001.

Motor performances of 3-, 5- and 9-month-old animals were tested in the accelerating rotarod, balance beam and inverted grid tasks (Figure 2). The accelerating rotarod task allows for the assessment of motor skill learning, motor coordination and balance. Already at 3 months of age, Tg4–42^hom^ and PS19/Tg4–42^hom^ displayed a deficit in the rotarod test with significantly reduced fall latencies compared to WT (*p* < 0.05) and PS19 (*p* < 0.001) groups (Figure 2a). At 5 months, these deficits remained consistent but significant differences were found only in comparison to the PS19 line (*p* < 0.05 for PS19/Tg4–42^hom^, *p* < 0.001 for Tg4–42^hom^) (Figure 2b). Tg4–42^hom^ and PS19/Tg4–42^hom^ performances in the accelerating rotarod task progressively worsened over time, with a further reduction in fall latencies at 9 months, resulting in significantly reduced values compared to both the WT and PS19 groups (*p* < 0.001) (Figure 2c). Young mice performed comparably in the balance beam task (Figure 2d), while at the 5-months’ time point, PS19/Tg4–42^hom^ displayed a deficit, presenting a significantly reduced latency to fall when compared to WT, PS19 and Tg4–42^hom^ mice (*p* < 0.001) (Figure 2e). Aged Tg4–42^hom^, in addition to PS19/Tg4–42^hom^ mice, showed a significantly reduced fall latency compared to WT and PS19 animals (Figure 2f), though no differences were observed between the two impaired lines. In the inverted grid task, no differences among the groups were revealed at any time point (Figure 2g–i).

### 2.3. Co-Expression of Aβ_4-42_ and Mutant Tau Leads to Spatial Memory Deficits in PS19/Tg4–42^hom^ Mice

**Figure 3:**
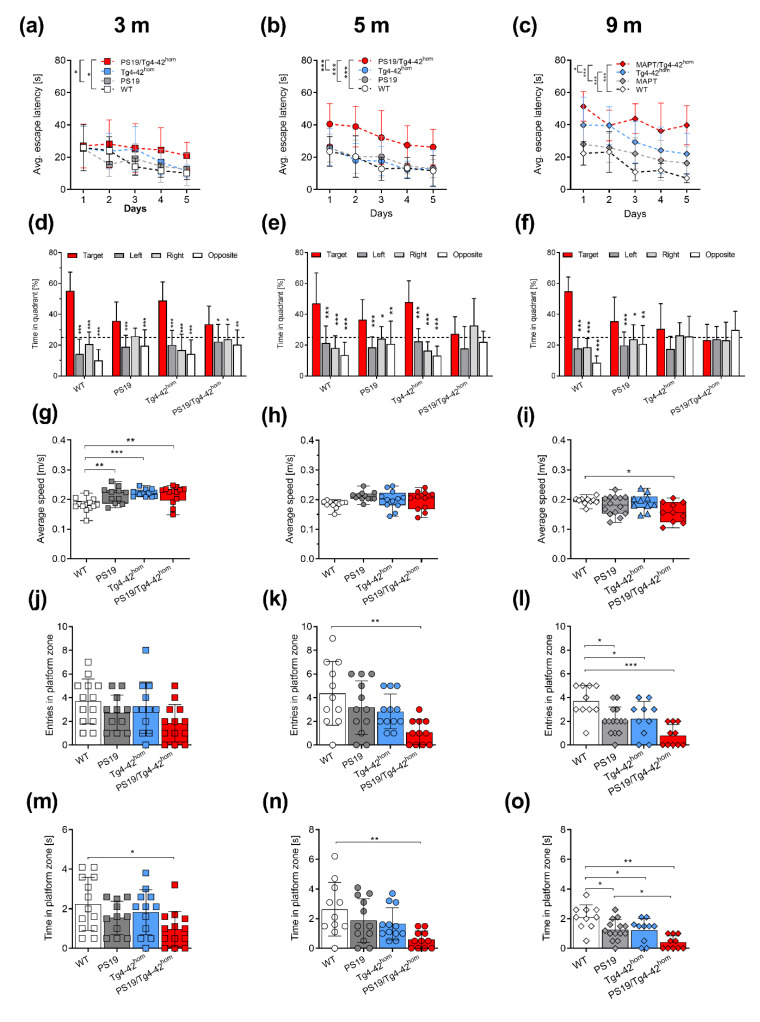
Spatial memory deficits in PS19/Tg4–42^hom^ mice. Female and male WT, PS19, Tg4–42^hom^ and PS19/Tg4–42^hom^ mice were tested in the Morris Water Maze test (*n* = 10–12) at 3 (**a**,**d**,**g**,**j**,**m**), 5 (**b**,**e**,**h**,**k**,**n**) and 9 (**c**,**f**,**i**,**l**,**o**) months of age. Spatial learning deficits were detected during the acquisition training in PS19/Tg4–42^hom^ already at 3 months (**a**), which worsened with aging (**b**,**c**). No spatial reference memory deficits were observed in young animals during the probe trial (**d**), while 5-month-old mice co-expressing Aβ_4-42_ and mutant human tau failed to remember the position of the goal quadrant (**e**). Aged Tg4–42^hom^ and PS19/Tg4–42^hom^ mice both displayed deficits in the probe trial (**f**). The transgenic lines swam significantly faster than WT littermates at young age (**g**), while no differences in average speed were observed at 5 months of age (**j**). Aged PS19/Tg4–42^hom^ swam significantly slower than WT controls (**i**). Compared to WT mice, PS19/Tg4–42^hom^ mice showed a reduced time in the platform zone at 3 (**m**) and 5 months (**l**), as well as a reduced number of platform zone entries at 5 (**k**) but not at 3 months of age (**j**). All three transgenic lines showed reduced entries and time spent in platform zone at 9 months (**l**, **o**). All data are expressed as mean ± SD. (**a**–**c**) Two-way RM ANOVA or (**d**–**f**) Two-way ANOVA followed by Bonferroni’s multiple comparison test. (**g**–**o**) One-way ANOVA followed by Bonferroni’s multiple comparison: * *p* < 0.05, ** *p* < 0.01, *** *p* < 0.001.

Spatial reference memory was assessed using the MWM task. During the cued training, all analyzed groups showed progressive decrease in escape latencies at 3 and 5 months of age, however, PS19/Tg4–42^hom^ displayed a significant increase in latency compared to Tg4–42^hom^ mice (*p* < 0.05) at 3 months, as well as to PS19 (*p* < 0.001) and WT (*p* < 0.01) mice at 5 months of age (Supplementary Figure S1). At 9 months of age, all three transgenic lines showed an increased escape latency compared to WT controls (PS19: *p* < 0.05, Tg4–42^hom^ and PS19/Tg4–42^hom^: *p* < 0.001). While the two parental lines presented a progressive decrease over the three days of testing, PS19/Tg4–42^hom^ mice displayed the poorest performance, significantly different from the two parental lines (*p* < 0.001) (Supplementary Figure S1). In the subsequent acquisition training, PS19/Tg4–42^hom^ mice already at 3 months presented a significantly increased escape latency compared to WT and PS19 mice (*p* < 0.05), indicative of the development of a spatial learning deficit in this line (Figure 3a). The deficits observed in PS19/Tg4–42^hom^ mice worsened over time, with a poorer performance at 5 months in the acquisition training and a significantly increased latency to reach the platform compared to all other investigated lines (*p* < 0.001) (Figure 3b). As expected, Tg4–42^hom^ mice displayed spatial learning deficits at 9 months, needing significantly more time to reach the hidden platform compared to WT mice (*p* < 0.001; Figure 3c). The spatial learning impairment observed in 5-month-old PS19/Tg4–42^hom^ mice worsened as time progressed, as the aged group presented an increased escape latency compared to both WT (*p* < 0.001) and the two parental lines (PS19: *p* < 0.001, Tg4–42^hom^: *p* < 0.05) (Figure 3c). Significantly different average speeds were noticed among the groups during both cued and acquisition trainings (Supplementary Figures S1 and S2). While the WT group was the one usually swimming most slowly at 3 and 5 months, at 9 months, Tg4–42^hom^ and PS19/Tg4–42^hom^ lines showed a reduced average speed in the cue training, but no differences were observed in the acquisition training session (Supplementary Figure S2).

In the probe trial, preference for the goal quadrant was analyzed to determine the presence of spatial reference memory deficits. All groups at 3 months showed a significant goal quadrant preference compared to the other quadrants (Figure 3d). Interestingly, this preference was not observed in 5-month-old PS19/Tg4–42^hom^ mice (Figure 3e), indicating a spatial reference memory deficit at this time point, which could not be explained by differences in swimming speed (Figure 3g,h). In the aged groups, both Tg4–42^hom^ and PS19/Tg4–42^hom^ mice displayed spatial memory deficits, swimming a comparable amount of time among the four quadrants, while PS19 and WT mice showed a preference for the goal quadrant (Figure 3f). At this time point, PS19/Tg4–42^hom^ mice swam significantly slower in the probe trial compared to WT mice (*p* < 0.05) (Figure 3i). A direct comparison of time spent in the goal quadrant during the probe trial among the groups revealed a significant reduction in PS19/Tg4–42^hom^ compared to WT (*p* < 0.001) and Tg4–42^hom^ (*p* < 0.05) mice, starting already at 3 months of age and persisting at 5 months, while no differences were observed compared to PS19 littermates (Supplementary Figure S3). At 9 months, all three transgenic lines spent significantly less time in the target quadrant compared to WT controls (Supplementary Figure S3). Additionally, PS19/Tg4–42^hom^ mice showed significantly less entries into the platform zone at 5 (Figure 3k) but not at 3 months (Figure 3j) and spent significantly less time in the platform zone during the probe trial compared to the WT group, both at 3 and 5 months (*p* < 0.05 and *p* < 0.01, respectively) (Figure 3m,n). Aged transgenic lines displayed reduced entries and time spent in the platform zone when compared to non-transgenic mice (Figure 3l,o), and PS19/Tg4–42^hom^ spent significantly less time in the platform zone than PS19 littermates (*p* < 0.05) (Figure 3o).

In contrast to spatial reference memory, no significant alteration was observed in PS19/Tg4–42^hom^ mice with regard to recognition memory (Supplementary Figure S4). In young animals, all transgenic lines showed a preference towards the novel object and showed comparable discrimination index (DI) values, although Tg4–42^hom^ DI resulted significantly lower than WT controls (*p* < 0.05), which might be attributed to the small sample variability. Though PS19/Tg4–42^hom^ mice showed the lowest DI of all groups at the 5-month time point, they did not perform significantly worse compared to the other genotypes. At 9 months, both Tg4–42^hom^ and PS19/Tg4–42^hom^ mice showed an impaired recognition memory, being unable to discriminate between the novel and familiar objects. These two lines showed a DI close to zero, and significantly lower than WT controls. Moreover, the PS19/Tg4–42^hom^ group showed a reduced DI compared to PS19 littermates (Supplementary Figure S4).

### 2.4. Increased Distal CA1 Neuron Loss in Young PS19/Tg4–42^hom^ Mice

**Figure 4:**
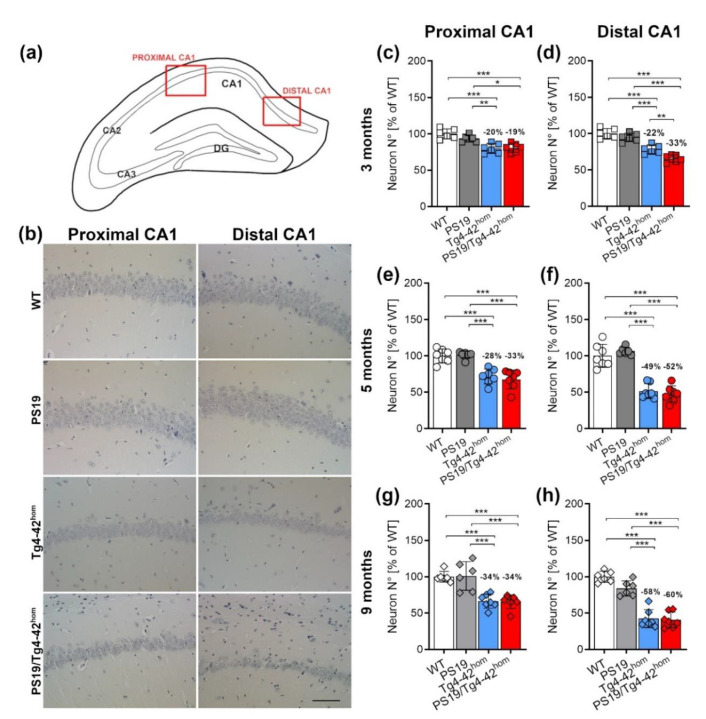
Increased distal CA1 neuron loss in young PS19/Tg4–42^hom^ mice. Sagittal paraffin brain sections (n = 3 per animal) from female and male WT, PS19, Tg4–42^hom^ and PS19/Tg4–42^hom^ mice (*n* = 6–7) were stained with hematoxylin. (**a**) Images were taken at 400x magnification from the distal and proximal part of the hippocampal CA1 pyramidal layer. (**b**) Example images of the CA1 pyramidal layer at 5 months of age. Tg4–42^hom^ and PS19/Tg4–42^hom^ mice displayed a comparable neuron loss at 5 and 9 months of age in both distal and proximal CA1 (**e–h**). In young PS19/Tg4–42^hom^ mice, a significant reduction in neuron numbers was observed compared to Tg4–42^hom^ in the distal (**d**), but not in the proximal CA1 area (**c**). All data are given as mean ± SD. One-way ANOVA followed by Bonferroni’s multiple comparison test: * *p* < 0.05, ** *p* < 0.01, *** *p* < 0.001. Scale bar: 50 μm.

**Figure 5:**
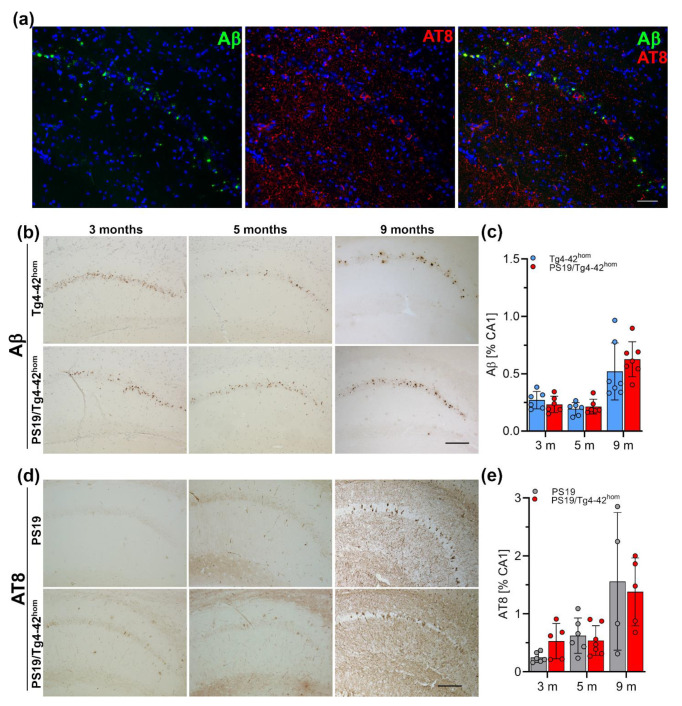
Phosphorylated tau and Aβ_4__–42_ immunoreactivity. (**a**) Fluorescent staining of Aβ_4–42_ (green) and phosphorylated tau protein (AT8—red) in the CA1 pyramidal layer from a 3-month-old PS19/Tg4–42^hom^ mouse did not reveal a major co-localization. Representative Aβ (**b**) and AT8 (**d**) staining in CA1 of PS19, Tg4–42^hom^ and PS19/Tg4–42^hom^ mice. Immunohistochemical analysis (n = 5–7 per time point, 3 sections per animal) showed no difference in Aβ immunoreactivity between Tg4–42^hom^ and PS19/Tg4–42^hom^ mice at any time point (**c**). A tendency towards an increase in the area covered with phosphorylated tau in 3-month-old PS19/Tg4–42^hom^ mice, while no differences in tau pathology were observed at 5 months between PS19 and PS19/Tg4–42^hom^ animals (**e**). All data are given as mean ± SD. Unpaired *t*-test. Scale bars: (**a**): 50 µm, (**b**, **d**): 100 μm.

Homozygous Tg4–42 mice display an age-dependent neuron loss in the CA1 region of the hippocampus, reaching a plateau after 6 months of age, a time point when deficits become evident in a variety of behavioral tasks [27]. Hematoxylin staining was performed to assess the effects of the co-expression of transgenic Aβ_4-42_ and mutant tau on CA1 neuron numbers in 3-, 5- and 9-month-old bigenic animals (Figure 4b and Supplementary Figure S5). The CA1 pyramidal layer was analyzed separately for the distal and proximal region, as these two areas are believed to be involved in different types of memory (Figure 4a). Tg4–42^hom^ mice presented the expected age-dependent neuron loss with a total CA1 (calculated as distal + proximal) neuron loss of approximately 20%, 40% and 45% compared to WT mice at 3, 5 and 9 months, respectively (Supplementary Figure S6), confirming previous data obtained with stereological analyses [27]. At the considered time points, the PS19 line did not show any difference compared to WT mice, neither in the distal nor proximal region of CA1 (Figure 4c–h), in good agreement with previous findings showing no hippocampal neuron loss in this model until 9–12 months [26]. In contrast, Tg4–42^hom^ and PS19/Tg4–42^hom^ mice displayed a significant age-dependent neuron loss in the distal as well as the proximal CA1 at both time points when compared to the WT and PS19 groups (*p* < 0.001) (Figure 4c–h). At all considered time points, the neuron loss is more prominent in the distal part (Figure 4b), the CA1 region where the Tg4–42^hom^ line preferentially accumulates Aβ_4-42_ peptides (Figure 5b). PS19/Tg4–42^hom^ mice, expressing both transgenic tau and Aβ_4–42_, presented an increased neuron loss compared to Tg4–42^hom^ animals (*p* < 0.01) in the distal part (Figure 4d and Supplementary Figure S5) but not in the proximal part of the CA1 region of the hippocampus at the age of 3 months (Figure 4c and Supplementary Figure S5). On the contrary, no difference in neuron numbers was observed between these two lines in either the distal or the proximal CA1 part at both the 5- and 9-month time points (Figure 4b,e–h and Supplementary Figure S5).

### 2.5. Hippocampal Aβ Ppathology and Tau Phosphorylation in PS19/Tg4–42^hom^ Mice

Fluorescent immunohistochemistry was performed on paraffin brain sections to investigate if Aβ_4–42_ and tau co-localize within the same neurons in PS19/Tg4–42^hom^ mice (Figure 5a). The pyramidal layer was analyzed specifically as it is the main region expressing Aβ_4–42_ in Tg4–42^hom^ mice. No major co-localizations of Aβ_4–42_ and tau immunoreactivity were observed within the same pyramidal neuron. Next, Aβ_4–42_ pathology was quantified in the CA1 region of the hippocampus and compared between Tg4–42^hom^ and PS19/Tg4–42^hom^ (Figure 5b,c) mice after staining with the 24311 antibody. No significant differences in Aβ load were detected in PS19/Tg4–42^hom^ compared to single transgenic Tg4–42^hom^ mice at either 3, 5 or 9 months of age (Figure 5c). To determine whether transgenic Aβ_4–42_ accumulation aggravates tau pathology, hyperphosphorylated tau, immunohistochemically stained with the AT8 antibody, was quantified in the CA1 region of the hippocampus comparing PS19 and PS19/Tg4–42^hom^ mice (Figure 5d,e). At 3 months of age, an increased immunoreactivity of phosphorylated tau was detected upon Aβ_4–42_ expression in PS19/Tg4–42^hom^ mice, with a trend towards statistical significance (*p* = 0.0573). In contrast, no significant difference was observed at 5 months of age between PS19 and PS19/Tg4–42^hom^ mice (Figure 5e). At 3 and 5 months of age, phospho-tau immunoreactivity presented mostly as a diffuse staining with very few intracellular accumulations. This was predominantly observed in the bigenic line, but without a clear aggravation in tau pathology. At 9 months, abundant tau pathology with NFTs could be observed in the CA1 pyramidal layer, but with no significant difference between PS19 and PS19/Tg4–42^hom^ mice.

## 3. Discussion

In general, PS19 mice did not show overt deficits compared to age-matched WT mice in terms of motor or memory performance at younger ages. With regard to the accelerating rotarod task, they even seemed to perform slightly better than WT littermates, however without reaching statistical significance. This is consistent with a hyperactivity phenotype in 6-month-old PS19 mice, presenting with better, albeit not statistically significant, performance on the rotarod compared to WT control animals [28]. A related observation was reported recently in 3.5- to 12-month-old PS19 mice, showing a ~10% prolonged latency in the accelerating rotarod test [29].

Potential effects of excess Aβ peptides on an already existing tau pathology have been studied in several experimental paradigms. A substantially increased number of NFTs was detected in P301L tau transgenic mice following injection of fibrillary Aβ_42_ peptides [30] and an induction of tau pathology was observed in the same line of tau transgenic mice after infusion of an Aβ-containing brain extract from a 24-month-old APP23 mouse [31]. Related findings were reported by Vasconselos and colleagues, showing that pre-aggregated Aβ is able to induce fibrillization of tau both in vitro and in vivo [32], and interestingly, passive immunization against Aβ in the 3xTg mouse model not only reduced extracellular amyloid plaques but also resulted in decreased tau pathology [33].

The lack of an influence of mutant tau on Aβ accumulation, as observed in the present report, confirms previous studies in APP/Tau transgenic lines with robust extracellular Aβ deposition. Using the same line of P301S tau transgenic mice, a trend towards a higher extracellular amyloid plaque load was detected in PDAPP/Tau bigenic mice [8], while no evidence for increased Aβ pathology was evident in 5XFAD/PS19 mice at either 3 or 9 months of age [9]. However, it has to be noted that an up to 5-fold increased amyloid load was observed in 16-month-old Tg2576 mice that have been crossed with a tau transgenic line harboring a triple-mutant tau (G272V, P310L, R406W) [34].

The Tg4–42 mouse model used in the present study primarily shows an intraneuronal accumulation of Aβ peptides, in particular in CA1 hippocampal neurons, and does not form overt extracellular plaques [21]. A related model expressing only Aβ_1–42_ peptides with the rat preproenkephalin signal peptide (APP48) in the absence of APP overexpression developed intracellular Aβ lesions and presented with reduced hippocampal neuron numbers already at young age [35]. This line had been crossed with tau transgenic mice overexpressing human 4-repeat tau with the P301S mutation (TAU58). Double transgenic mice showed neither evidence of increased levels of soluble Aβ, albeit data on Aβ levels in single transgenic APP48 mice were not reported, nor significant amounts of phospho-tau pathology [20]. Similarly, we observed no significant increase of phosphorylated tau in PS19/ Tg4–42^hom^ mice except for a trend in young animals. These findings in models harboring transgenic Aβ peptides in the absence of APP overexpression strikingly contrast the obvious exacerbation of tau pathology observed in tau transgenic mice when crossed with mouse models of AD overexpressing mutant APP [7,8,9,10,11,12,13,36]. The overexpression of APP in the studied mouse models could be a determining factor in the exacerbation of tau pathology. Takahashi and colleagues [37] showed that the presence of APP induced intracellular phosphorylated tau aggregation in cell culture exposed to tau fibrils in a dose-dependent manner. In addition, recent data suggested that the toxic effects of oligomeric tau on memory and long-term potentiation (LTP) in WT mice appear to be APP-dependent [38].

With regard to hippocampal neuron loss, a significantly decreased number of CA1 pyramidal cells has been detected in 9-month-old 5XFAD/PS19 mice in comparison to their parental single transgenic 5XFAD or PS19 lines [9]. A related observation was made in 3-month-old PS19/Tg4–42^hom^ mice, showing a significantly reduced CA1 pyramidal cell number in the distal part of the CA1 region. The lack of such a difference in 5-month-old mice might be attributed to the fact that single Tg4–42^hom^ mice already show a profound CA1 neuron loss, reaching a kind of plateau at that time point [27]. Indeed, between 5 and 9 months, the neuron loss in Tg4–42^hom^ mice worsens by less than 10%. While the distal part of the CA1 region appears to be mainly involved in non-spatial memory, the proximal part is supposed to play a more important role in tasks depending on spatial information [39,40]. Though Tg4–42^hom^ as well as PS19/Tg4–42^hom^ mice showed a significant loss of distal CA1 pyramidal neurons already at 3 and 5 months, this is not reflected in a major impairment of recognition memory, at least not detectable in the NOR task carried out in the present study. A deficit in this task became obvious in aged mice, which was accompanied by the loss of ~60% of neurons in the distal CA1. Neuron loss in the proximal CA1 part was most pronounced in 5-month-old PS19/Tg4–42^hom^ mice. At this time point, bigenic mice showed spatial memory deficits in the MWM task, however, it has to be noted that neuron numbers did not differ significantly from single transgenic Tg4–42^hom^ mice still learning the task, confirming results from a previous study employing 5-month-old Tg4–42^hom^ mice [27]. By 9 months of age, Tg4–42^hom^ mice display obvious spatial learning deficits. Despite comparable loss of proximal pyramidal neurons with Tg4–42^hom^ at this time point, PS19/Tg4–42^hom^ displayed the worst performances in goal quadrant and platform parameters among the transgenic lines. The presence of spatial memory deficits in the PS19 line is controversial, with some studies pointing towards the development of deficits in the MWM test from a relatively young age of 5 [28] or 6.5 months [41], while others report initial deficits in spatial memory at 10 [42] or even 12 months of age [29], respectively. The tau transgenic line did not present with loss of CA1 pyramidal neurons at the considered time points, but neuron loss in the CA3 and reduced DG volume have been described in P301S mice starting at 8 months [26,43].

Although the distal CA1 neuron loss observed in young PS19/ Tg4–42^hom^ mice was accompanied by a tendency towards increased phospho-tau immunoreactivity, no obvious behavioral alterations were observed at this time point, while in aged mice, the behavioral deficits observed in the bigenic line could not be related to an aggravation of neuron loss or tau pathology. The absence of obvious aggravation of tau pathology might suggest that the co-presence alone of Aβ_4–42_ and transgenic human tau and their additive singular detrimental effects could lead to the observed phenotypes in young mice. As an aggravation of tau pathology has been reported repeatedly in a variety of APP/tau transgenic lines, our data might support an important confounding role of transgenic human APP overexpression.

## 4. Materials and Methods

### 4.1. Transgenic Mice

The generation of the Tg4–42 line was previously described [21]. In brief, the human Aβ_4-42_ sequence is fused to the signal peptide sequence of the thyrotropin-releasing hormone and the expression is under the control of the Thy1 promoter. The Tg4–42 mice were bred to homozygosity (Tg4–42^hom^). The line was generated and maintained on a C57BL/6J genetic background.

PS19 mice overexpress human tau with the P310S mutation under the control of the murine prion protein promoter [26] and were purchased from Jackson laboratories (Bar Harbor, ME, USA) (B6;C3-Tg(Prnp-MAPT*P301S)PS19Vle/J). Mice were backcrossed to C57Bl/6J for more than 5 generations.

Bigenic mice (PS19/Tg4–42^hom^) were generated by breeding transgene-positive Tg4–42^hom^ and PS19 mice and were maintained on a C57BL/6J genetic background. Accordingly, littermates were only obtained for Tg4-42^hom^ and PS19/Tg4-42^hom^ (with an additional tau transgene). In a second line of breeding, WT mice were bred with heterozygous PS19 mice to obtain WT and PS19 littermates. An equal number of female and male transgenic and age-matched C57Bl/6J (WT) mice at 3, 5 and 9 months of age were used in the present study. All animals were handled according to the German guidelines for animal care and all experiments have been approved by the local animal care and use committee (LAVES, Lower Saxony, Germany). Food and water were provided ad libitum.

### 4.2. Reverse Transcription and qRT-PCRs

Deep-frozen hippocampus samples were used to isolate mRNA. The tissues were weighted, supplied with 1 mL of Trifast^®^ reagent (PegLab, Wilmington, DE, USA) per 100 mg of sample and homogenized using a glass-Teflon homogenizer with 15 strokes at 800 rpm. RNA isolation was performed following the manufacturer’s protocol. RNA samples were subjected to digestion with DNAse I, followed by reverse transcription using the RevertAid RT Kit (Thermo Fisher Scientific, Waltham, MA, USA) according to the protocol of the supplier. The Biozym Blue S’Green qPCR Mix, containing SYBR Green as the intercalating fluorescent dye, was used to perform gene expression analysis. Raw data were collected using the MxPro Mx3000P software (Stratagene, Bellingham, WA, USA) and the average Ct value was calculated from the duplicate for each sample. The relative expression of the genes of interest (GOIs) was performed using murine β-Actin as a reference gene for normalization, and were calibrated to a selected control group using the ΔΔCt method [44]. The following primer sets were employed, as in Table 1.

### 4.3. Behavioral Tasks

#### 4.3.1. Accelerating Rotarod

A computer-controlled rotarod system (TSE, Technical and Scientific Equipment) was used to assess motor performance and motor learning in the rotarod task [45]. During 2 days of testing, each animal performed 4 trials, at least 10 min apart, per day. In each trial, the rod accelerated from 4 to 40 rpm over a maximum trial time of 300 s and the latency to fall was recorded. The task was performed under red light condition and the apparatus was cleaned with 70% ethanol between each trial to avoid odor cues.

#### 4.3.2. Balance Beam

In order to assess balance and fine motor coordination, the balance beam test was conducted [46]. Mice were placed on the center of a wooden beam (1 cm wide, 50 cm long, 44 cm high), at both ends of which a 9 × 15 cm escape platform was attached. The ground surface underneath the beam was padded to avoid possible injures. Each mouse performed three consecutive 60 s trials with at least 10 min intervals in between in one single day of testing. The latency to fall from the beam was recorded, while if a mouse remained on the beam for the whole 60 s trial or escaped to one of the platforms, the maximum time of 60 s was given.

#### 4.3.3. Inverted Grid

Neuromuscular abilities, vestibular function and muscle strength were tested with the inverted grid task [47]. Each mouse was positioned in the center of a metallic wire grid (45 cm in length, 30 cm in width, with a grid spacing of 1 cm), which was inverted and suspended 40 cm above a padded surface. The latency to fall was recorded during a single 60 s trial.

#### 4.3.4. Morris Water Maze (MWM)

The Morris Water Maze (MWM) was used to assess spatial reference memory [48] and was performed as previously described [21]. In brief, mice were at first subjected to 3 days of cued training, each consisting of four 60 s trials 10 min apart. During each trial of the cue training, the platform was marked with a triangular flag. The location of the platform and the starting point for the mice always changed between the four quadrants. A 5-day acquisition training (4 trials/day) followed 24 h after the end of the cue training. In this phase, the flag was removed from the platform that remained stationary for each mouse and in each trial. Proximal and distal visual cues were present during the acquisition training. During both cue and acquisition training, the latency to reach the platform, average speed and distance were recorded with an automated video tracking system (ANY-Maze, Stoelting, Wood Dale, IL, USA). Twenty-four hours after the last day of acquisition training, a probe trial was performed to address spatial memory. The platform was removed from the pool and mice were introduced from a novel entry point. Mice were allowed to freely swim for the 60 s trial duration and abidance in the different quadrants was recorded.

#### 4.3.5. Novel Object Recognition (NOR)

The novel object recognition (NOR) task, based on the innate preference of rodents for novelty, was performed to test for recognition memory. During the first day of testing, two identical objects were placed in the arena and presented to the mice, which could freely explore during a single trial session. On the second day of testing, one of the identical objects was exchanged to a novel object. On both days, exploration time of each object was recorded manually for every mouse during the single 5 min trial sessions. The recognition performances was quantified using the Discrimination Index (DI), measured as the differences between novel (T_novel_) and familiar (T_familiar_) object exploration times in proportion to the animal’s total exploration time (T_total_) [25].

### 4.4. Tissue Collection and Preservation

Mice were deeply anesthetized through an intraperitoneal injection of a mixture of ketamine and xylazine and were transcardially perfused using ice-cold 0.01 M phosphate-buffered saline (PBS). The brains were rapidly and carefully removed from the skull. The right hemisphere was drop-fixed in 4% formalin solution at 4 °C for at least 72 h protected from light and subsequently embedded in paraffin. The left hemisphere was dissected to obtain hippocampi samples that were deep-frozen on dry-ice and stored at −80 °C until processing.

### 4.5. Quantification of CA1 Neuron Number

Neuron loss was assessed in the CA1 region of the hippocampus on sagittal brain sections (bregma 0.72–1.08) of 3- and 5-month-old mice (n = 6–7 per time point) as previously described [25]. Paraffin sections of 4 µm thickness (3 sections per animal, at least 30 μm apart) were stained with hematoxylin to identify the nuclei. Neuronal nuclei were distinguished from glia cells based on their size and characteristic appearance. Images from the distal (towards subiculum) and proximal (extending to CA2) part of the CA1 were acquired using an Olympus BX-51 microscope equipped with a Moticam pro 282 camera (Motic, Wetzlar, Germany) at 400× magnification. CA1 pyramidal neurons in a defined area were counted using the manual cell counting tool implemented in ImageJ1.51 (NIH, Bethesda, MD, USA).

### 4.6. Immunohistochemistry

Sagittal paraffin brain samples were cut at 4 μm and used in immunohistochemical staining. Sections were processed as previously described [49]. In brief, paraffin was removed, incubating the slides in xylol and the sections were rehydrated with an ascending ethanol series. Endogenous peroxidases were blocked with a 30 min treatment of 0.3% H_2_O_2_ in 0.01 M phosphate-buffered saline (PBS), and antigens were retrieved by boiling sections in 0.01 M citrate buffer (pH 6.0). In case of amyloid-β staining, the epitopes were exposed to an additional 3 min treatment with 88% formic acid. An incubation of 4% skim milk in 0.01M PBS with 10% fetal cow serum (FCS) was applied for 1 h to block unspecific binding sites. The following primary antibodies, diluted to the desired concentration in 0.01 M PBS including 10% FCS, were applied overnight at room temperature in a humid chamber: 24311 (pan-Aβ, 1:500, rabbit pAb [9]) and AT8 (phosphorylated Tau pSer202/pThr205, 1:500, mouse mAb, Thermo Fisher Scientific, Dreieich, Germany). Biotinylated secondary antibodies (1:200, Dianova, Hamburg, Germany) or fluorescent-labelled secondary antibodies (1:750, Thermo Fisher Scientific, Dreieich, Germany) were applied for 1 h. Staining was visualized via the ABC method using the Vectastain kit (Vector Laboratories, Burlingame, CA, USA) with diaminobenzidine (DAB) as a chromogen and hematoxylin counterstaining. When a fluorescent immunohistochemistry was performed, 4’,6-diamidin-2-phenylindol (DAPI) was used to label the nuclei. Fluorescent images were taken using a Nikon TiE microscope (Nikon, Tokyo, Japan) and analyzed with NIS Elements imaging software (Nikon, Tokyo, Japan).

### 4.7. Quantification of Aβ and Tau Immunoreactivity

Serial images of the CA1 region of the hippocampus from DAB-stained sections were taken (n = 3 sections per animal), at least 30 μm apart from each other, using an Olympus BX-51 microscope equipped with a Moticam pro 282 camera (Motic, Wetzlar, Germany) with a 100x magnification lens (*n* = 5–6 per time point). The captured images were analyzed using the ImageJ software. A fixed intensity threshold was applied to define the DAB staining, after binary transformation to 8-bit black and white images. The percentage of the area covered by DAB staining was measured and compared between the different genotypes. Aβ load (24311) was quantified in Tg4–42^hom^ and PS19/Tg4–42^hom^, and tau immunoreactivity (AT8) in PS19 and PS19/Tg4–42^hom^ mice.

### 4.8. Statistical Analyses

All data have been analyzed using the Shapiro–Wilk test for normality to ensure that parametric tests can be applied. When parametric testing was possible, differences between groups were tested with unpaired *t*-test, one-way analysis of variance (ANOVA) followed by Bonferroni’s post-hoc, or repeated measure two-way ANOVA followed by Bonferroni’s post-hoc, as indicated. In case of non-parametric testing, the Mann–Whitney test was performed. All data were presented as means ± standard deviation (SD). Significance levels were as follows: * *p* < 0.05, ** *p* < 0.01, *** *p* < 0.001. All calculations were performed using GraphPad Prism version 8 for Windows (Graph Pad Software, San Diego, CA, USA).

## 5. Conclusions

As it has been shown previously that the presence of Aβ peptides leads to an aggravation of an existing tau pathology in a variety of transgenic AD mouse models, we evaluated the impact of soluble N-terminally truncated Aβ4-42 peptides in the widely used PS19 mouse model of AD. Though we detected a partial worsening of motor and spatial memory performance, as well as an aggravated CA1 neuron loss at young age, we did not observe the accelerated formation of tau aggregates reported in models with overt extracellular Aβ plaque pathology. Hence, the presence of extracellular amyloid plaques might be a prerequisite for enhanced neurofibrillary tangle formation in transgenic mice co-expressing human mutant APP and Tau.

## Figures and Tables

**Table 1:** Sequences of Forward and Reverse primers and probes used in qRT-PCR analyses.

Transgene/Gene	Forward Primer	Reverse Primer
Human *Aβ_4_*_–*42*_	TCCGGCCAGAACGTCGATTC	GGAGAAGCAAGACCTCTG
Human *MAPT*	CCAAGTGTGGCTCATTAGGCA	CCAATCTTCGACTGGACTCTGT
Murine *β-Actin*	ATGGAGGGGAATACAGCCC	ATGGAGGGGAATACAGCCC

## Data Availability

Original data is available from the authors upon reasonable request.

## Supplementary Materials

The following are available online at https://www.mdpi.com/article/10.3390/ijms22105191/s1, Figure S1: Average escape latency and speed in cued training of MWM, Figure S2: Water Maze—Speed in acquisition training, Figure S3: Water Maze—Time in target quadrant, Figure S4: Novel object recognition task, Figure S5: Examples of proximal and distal CA1, Figure S6: Neuron loss in total CA1.
