# Supplementary material for: Characterization of a Mouse Model of Alzheimer’s Disease Expressing Aβ4-42 and Human Mutant Tau
Source: Int J Mol Sci. 2021 May 14;22(10):5191. doi: 10.3390/ijms22105191 (PMC8156793; doi:10.3390/ijms22105191)
Supplement: Supplementary file 1 [file ijms-22-05191-s001.zip › ijms-1210297-supplementary.pdf]

Supplementary Material

Zampar S. & Wirths O.

Characterization of a mouse model of Alzheimer's disease expressing Aβ4-42 and human mutant tau

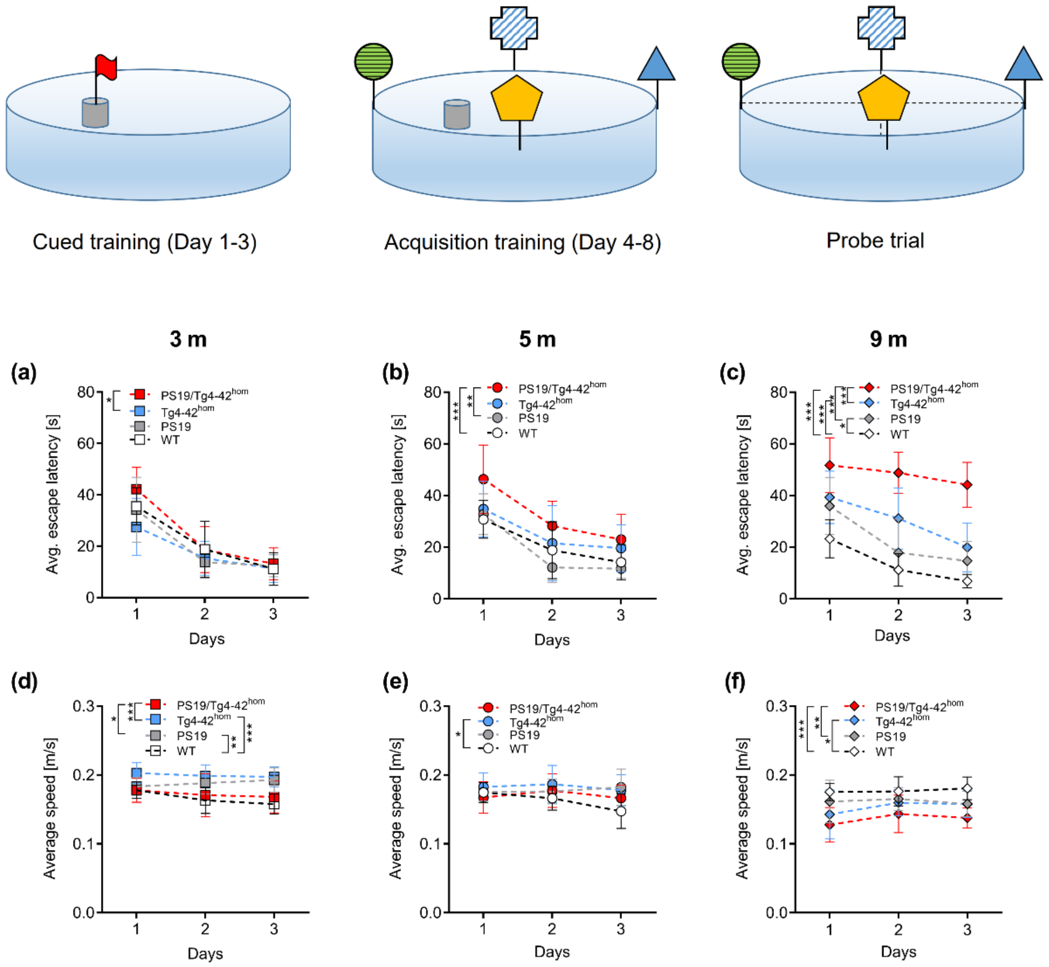

Supplementary Figure S1: Average escape latency and speed in cued training of MWM**.** The top panel illustrates the experimental setting used in the MWM. Female and male WT, PS19, Tg4-42^{hom} and PS19/Tg4-42^{hom} mice were tested in the Morris Water Maze test (n=10-12) at 3 (**a**,**e**), 5 (**b**,**e**) and 9 months (**c**,**f**) of age. All data are given as means ± SD. Two-way ANOVA RM, followed by Bonferroni's multiple comparison test: $*p < 0.05$, $**p < 0.01$, $***p < 0.001$

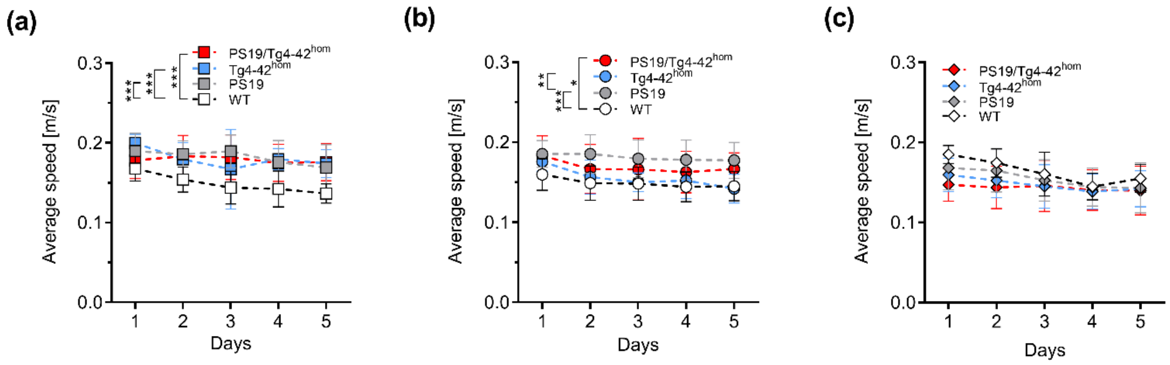

Supplementary Figure S2: Average speed in acquisition training of MWM. Female and male WT, PS19, Tg4-42^{hom} and PS19/Tg4-42^{hom} mice were tested in the Morris Water Maze test (n=10-12) at 3 (**a**), 5 (**b**) and 9 months (**c**) of age. All data are given as means ± SD. Two-way ANOVA RM, followed by Bonferroni's multiple comparison test: ***$p < 0.001$

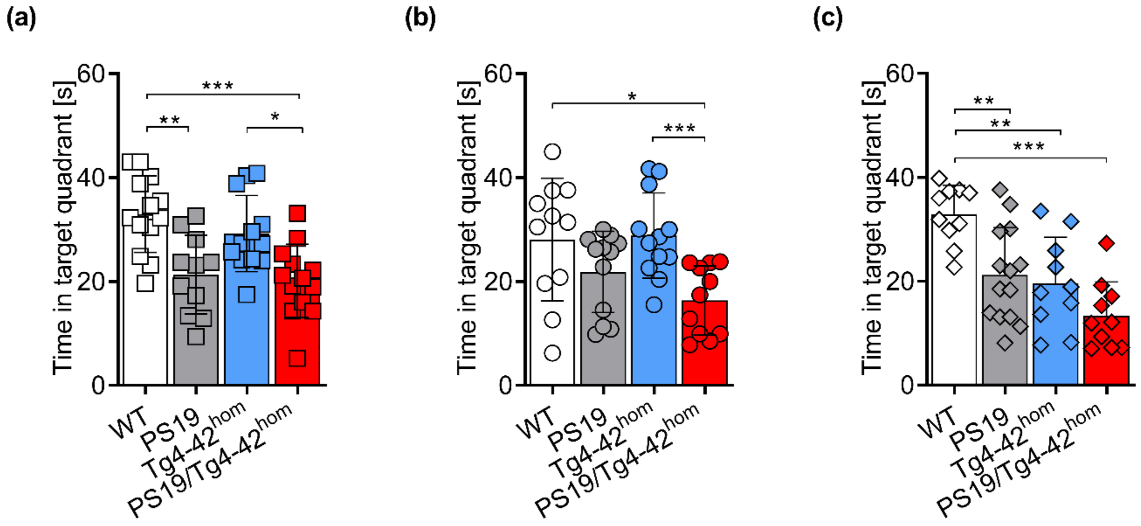

Supplementary Figure S3: Direct comparison of the time spent in the target quadrant in the probe trial of the MWM at 3 (**a**), 5 (**b**) and 9 (**c**) months of age. All data are given as means ± SD. One-way ANOVA followed by Bonferroni's multiple comparison test: *$p < 0.05$, **$p < 0.01$, ***$p < 0.001$.

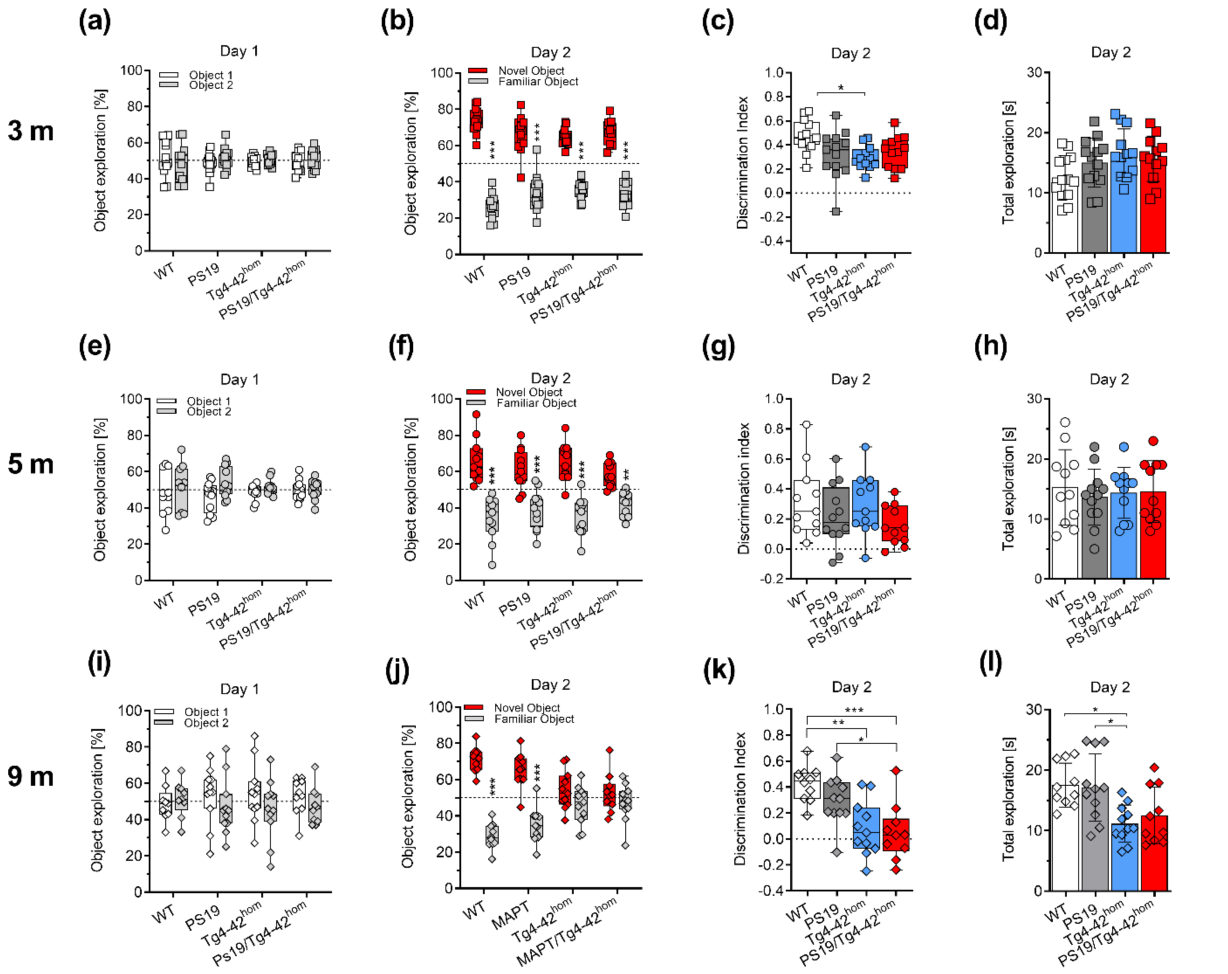

Supplementary Figure S4: Novel object recognition task. Female and male WT, PS19, Tg4–42^{hom} and PS19/Tg4–42^{hom} mice (n=10–14) were tested at 3 (**a–d**), 5 (**e–h**) and 9 (**i–l**) months. Training trial on day 1 showed no difference in exploration between the two identical objects in all groups at the considered time points (**a,e,i**). On the testing day, no recognition memory deficits were observed at 3 and 5 months in WT and the three transgenic lines (**b,f**), as they all significantly explored more the novel object compared to the familiar one. At 9 months, recognition memory deficits could be observed in Tg4–42^{hom} and PS19/Tg4–42^{hom} mice (**j**). Discrimination indices (DIs) were comparable at 3 and 5 months (**c,g**), only showing a significance reduction on young Tg4–42^{hom} due to small sample variability. At 9 months, both Tg4–42^{hom} and PS19/Tg4–42^{hom} showed significantly reduced DIs compared to WT, as well asPS19/Tg4–42^{hom} compared to PS19 (**k**). Total exploration time in the test trial on day 2 was measured and, while no differences were observed at 3 and 5 months, Tg4–42^{hom} mice displayed a significantly reduced exploration time at the 9 months' time point (**d,h,l**). All data are given as means ± SD. (**a,b,e,f,g,h**) Dotted line represents 50%, Two-way ANOVA followed by Bonferroni's multiple comparison test; (**c,d,g,h,k,l**) One-way ANOVA followed by Bonferroni's multiple comparison test: *p<0.05, **p<0.01, ***p<0.001.

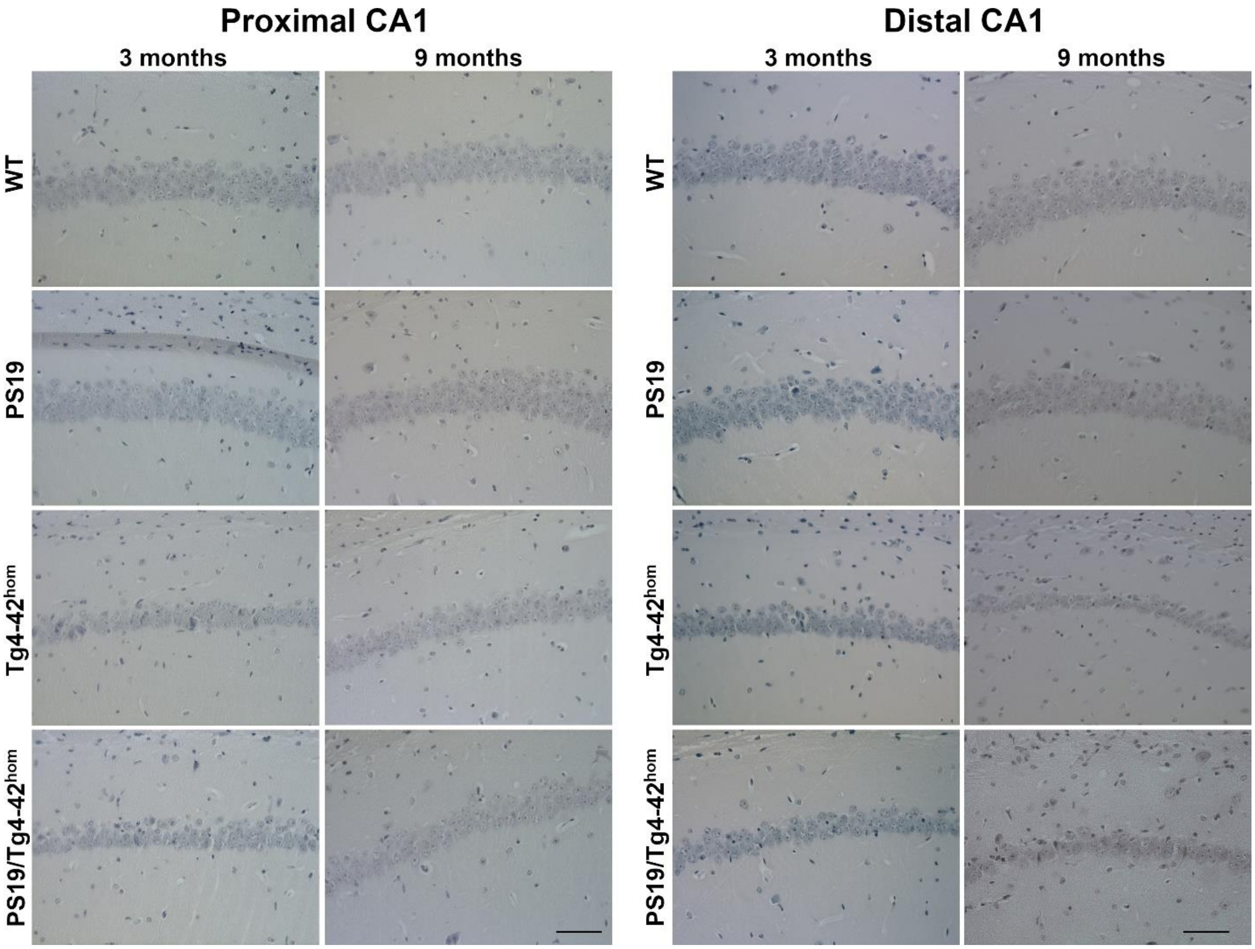

Supplementary Figure S5: Example images of proximal and distal CA1 pyramidal neurons at 3 and 9 months. Sagittal brain sections were stained with hematoxylin. Pictures were taken at 400-x magnification. Scale bar 50 µm.

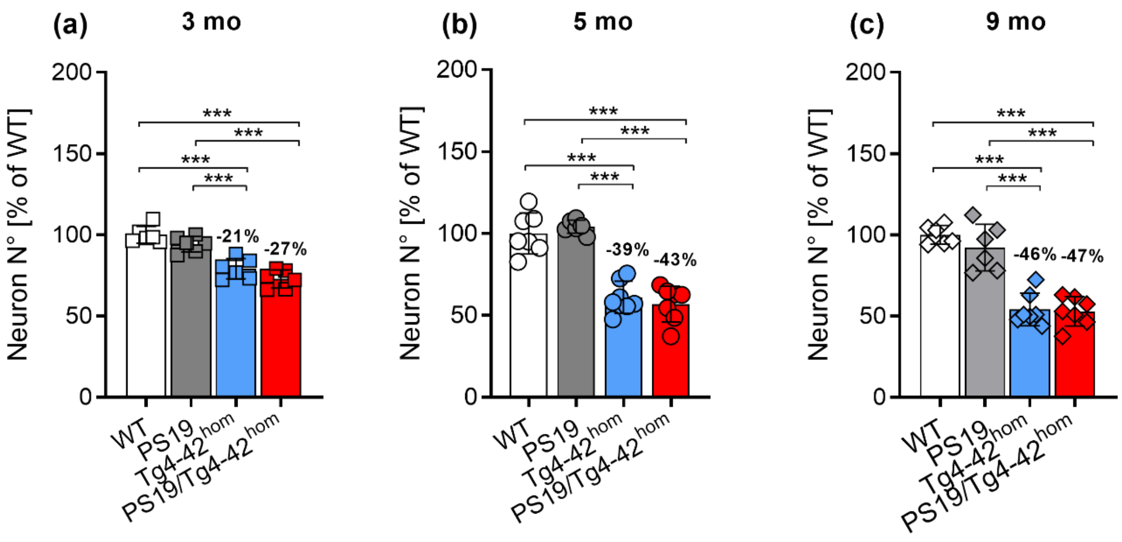

Supplementary Figure S6: Neuron loss in total CA1. Sagittal paraffin brain sections (n = 3 per animal) from female and male WT, PS19, Tg4–42^{hom} and PS19/Tg4–42^{hom} mice (n = 6 - 7) were stained with hematoxylin. The sum of distal and proximal CA1 regions is referred as total CA1. Significant neuron loss in Tg4–42^{hom} and PS19/Tg4–42^{hom} mice at 3 months (**a**), 5 (**b**) and 9 months (**c**). All data are given as mean ± SD. One-way ANOVA followed by Bonferroni's multiple comparison test: ***$p < 0.001$.
